# Supplementary material for: Mechanical Force-Induced Blue-Shifted and Enhanced Emission for AIEgens
Source: Biosensors (Basel). 2022 Nov 21;12(11):1055. doi: 10.3390/bios12111055 (PMC9688405; doi:10.3390/bios12111055)
Supplement: Supplementary file 1 [file biosensors-12-01055-s001.zip › biosensors-1970552-supplementary.pdf]

Supporting Information

Mechanical Force-Induced Blue-Shifted and Enhanced Emission for AIEgens

Chang-Sheng Guo [1,†], Xiao-Long Su [1,†], Yu-Ting Yin [1], Bo-Xuan Zhang [1], Xin-Yi Liu [1], Rui-Peng Wang [1], Pu Chen [1], Hai-Tao Feng [1,*] and Ben-Zhong Tang [2,*]

[1] AIE Research Center, Shaanxi Key Laboratory of Phytochemistry, College of Chemistry and Chemical Engineering, Baoji University of Arts and Sciences, Baoji 721013, China

[2] Shenzhen Institute of Molecular Aggregate Science and Engineering, School of Science and Engineering, The Chinese University of Hong Kong, Shenzhen 518172, China

* Correspondence: haitaofeng907@163.com (H.-T.F.); tangbenz@cuhk.edu.cn (B.Z.T.)

† These authors contributed equally to this work.

1. General information

Materials: All reagents and solvents were chemical pure (CP) grade or analytical reagent (AR) grade and were used as received unless otherwise indicated.

Measurements: ^{1}H NMR and ^{13}C NMR spectra were obtained by an Agilent NMR Systems 400MHz NMR Spectrometer at 298 K. High resolution mass spectra (HRMS) were obtained by use of a Bruker Compact TOF mass spectrometer in electrospray ionization mode (ESI+). Absorption spectra were recorded on a YOKU INSTRUMENT TS2023 UV-Vis spectrophotometer. Solid absorption spectra were recorded on a Perkin Elmer Lambda 1050+ spectrophotometer. Fluorescence spectra were collected on a HORIBA FLOUROMAX-4 fluorophotometer at 298 K. Powder X-ray diffraction (PXRD) was collected on Rigaku DMAX U1TlMAIV diffractometer using CuKα irradiation. The lifetimes were measured on an Edinburgh FLS1000 fluorescence spectrophotometer equipped with a continuous xenon lamp (Xe1). The surface morphology of the samples was analyzed using scanning electron microscope (SEM, FEI Quanta FEG 250). Single crystal data were collected on a Bruker Smart APEXII CCD diffractometer using graphite monochromated Mo Kα radiation (λ = 0.71073 Å) or Cu Kα radiation (λ = 1.54184 Å).

Computational details

Frontier molecular orbitals were calculated by Gaussian 09 software using density functional theory at B3LYP/6-311G* level.

2. General procedure for the synthesis of cyanostilbene–based AIEgens 1-6.

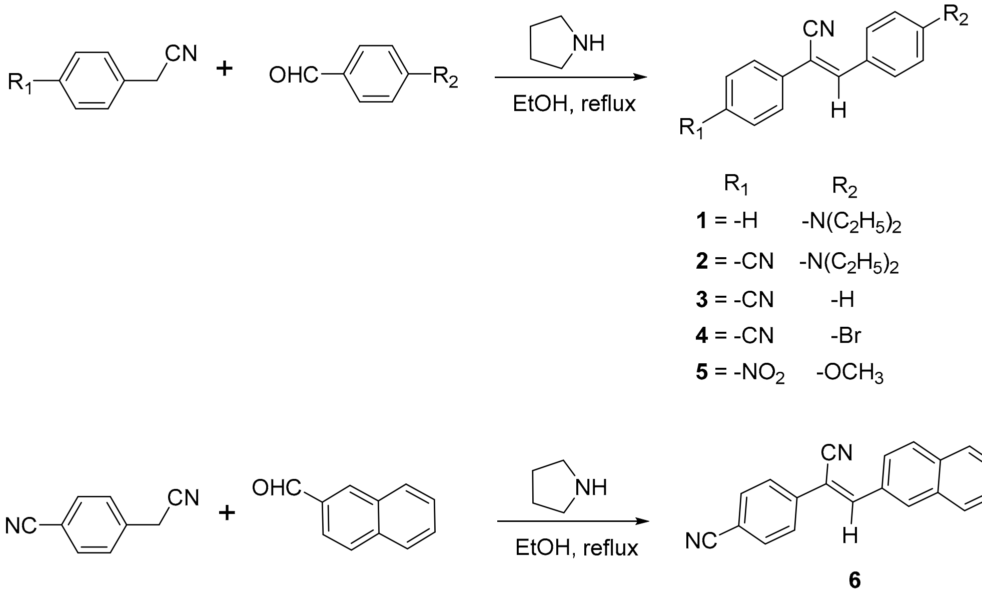

Scheme S1. The synthetic procedure of molecules **1-6**.

An appropriate benzeneacetonitrile (3 mmol) and benzaldehyde (3 mmol) and pyrrolidine (1~3 drops) were dissolved in toluene (15 mL) and the mixture was stirred at 80 °C for 6 h, and then cooled to 25 °C. The crude product was obtained by filtration. Finally, the crude product was recrystallized in DCM/ethanol mixture (v/v = 1/1) to give the corresponding product.

According to the general procedure, the product **1** was obtained in (0.66 g, yield 80%). ^{1}H NMR (400 MHz, $CDCl_3$) δ = 7.85 (d, J = 9.2 Hz, 2 H), 7.65 - 7.62 (m, 2 H), 7.43 - 7.40 (m, 3 H), 7.33 - 7.29 (m, 1 H), 6.70 (d, J = 9.2 Hz, 2 H), 3.43 (q, J = 7.2 Hz, 4 H), 1.22 (t, J = 8.2 Hz 6 H). ^{13}C NMR (100 MHz, $CDCl_3$) δ 149.50, 142.65, 135.85, 131.74, 128.98, 127.93, 125.53, 120.99, 119.72, 111.24, 103.90, 44.64, 12.74. ESI$^+$ HRMS m/z calcd. for $C_{19}H_{21}N_2$ 277.1699 $[M+H]^+$, found 277.1697 $[M+H]^+$.

According to the general procedure, the product **2** was obtained in (0.74 g, yield 82%). ^{1}H NMR (400 MHz, $CDCl_3$) δ 7.87 (d, J = 9.2 Hz, 2 H), 7.71 (d, J = 8.8 Hz, 2 H), 7.66 (d, J = 8.8 Hz, 2 H), 7.46 (s, 1 H), 6.70 (d, J = 9.2 Hz, 2 H), 3.45 (q, J = 7.2 Hz, 4 H), 1.23 (t, J = 7.2 Hz, 6 H). ^{13}C NMR (100 MHz, $CDCl_3$) δ 150.15, 144.64, 140.28, 132.55, 132.35, 125.55, 120.13, 118.83, 118.63, 111.17, 110.74, 101.22, 44.57, 12.55. ESI$^+$ HRMS m/z calcd. for $C_{20}H_{20}N_3$ 302.1652 $[M+H]^+$, found 302.1644 $[M+H]^+$.

According to the general procedure, the product **3** was obtained in (0.60 g, yield 81%).

^{1}H NMR (400 MHz, $CDCl_3$) δ 7.94 – 7.91 (m, 2 H), 7.81 – 7.78 (m, 2 H), 7.76 – 7.73 (m, 2 H), 7.63 (s, 1 H), 7.51 – 7.48 (m, 3 H). ^{13}C NMR (100 MHz, $CDCl_3$) δ 145.03, 138.94, 133.12, 132.97, 131.68, 129.79, 129.31, 126.69, 118.31, 117.28, 112.89, 110.10. ESI$^+$ HRMS m/z calcd. for $C_{16}H_{10}N_2Na$ 253.0736 $[M+Na]^+$, found 253.0736 $[M+Na]^+$.

According to the general procedure, the product **4** was obtained in (0.85 g, yield 92%). ^{1}H NMR (400 MHz, $CDCl_3$) δ 7.80 – 7.77 (m, 4 H), 7.76 – 7.73 (m, 2 H), 7.65 – 7.61 (m, 2 H), 7.56 (s, 1 H). ^{13}C NMR (100 MHz, $CDCl_3$) δ 143.50, 138.58, 133.02, 132.62, 131.94, 131.07, 126.70, 126.21, 118.21, 117.03, 113.13, 110.77. ESI$^+$ HRMS m/z calcd. for $C_{16}H_9BrNa$ 330.9841 $[M+Na]^+$, found 330.9841 $[M+Na]^+$.

According to the general procedure, the product **5** was obtained in (0.80 g, yield 95%). ^{1}H NMR (400 MHz, $CDCl_3$) δ 8.28 (d, J = 8.8 Hz, 2 H), 7.94 (d, J = 8.8 Hz, 2 H), 7.81 (d, J = 8.8 Hz, 2 H), 7.60 (s, 1 H), 7.01 (d, J = 8.8 Hz, 2 H), 3.89 (s, 3 H). ^{13}C NMR (100 MHz, $CDCl_3$) δ 162.56, 147.66, 145.14, 141.23, 132.07, 126.47, 125.83, 124.44, 117.85, 114.80, 106.33, 55.68. ESI$^+$ HRMS m/z calcd. for $C_{16}H_{12}O_3Na$ 303.0740 $[M+Na]^+$, found 303.0739 $[M+Na]^+$.

According to the general procedure, the product **6** was obtained in (0.67 g, yield 80%).^{1}H NMR (400 MHz, $CDCl_3$) δ 8.33 (s, 1 H), 8.12 – 8.10 (dd, J = 8.8, 2.0 Hz, 1 H), 7.95 – 7.92 (d, J = 9.2 Hz, 2 H), 7.89 – 7.87 (m, 1 H), 7.84 – 7.81 (m, 2 H), 7.77 (s, 1 H), 7.76 – 7.73 (m, 2 H), 7.62 – 7.54 (m, 2 H). ^{13}C NMR (100 MHz, $CDCl_3$) δ 144.97, 139.07, 134.69, 133.15, 132.96, 131.57, 130.63, 129.15, 129.09, 128.41, 127.98, 127.23, 126.65, 125.19, 118.34, 117.50, 112.80, 109.87. ESI$^+$ HRMS m/z calcd. for $C_{20}H_{12}N_2Na$ 303.0893 $[M+Na]^+$, found 303.0889 $[M+Na]^+$.

3. Characteristic Spectra

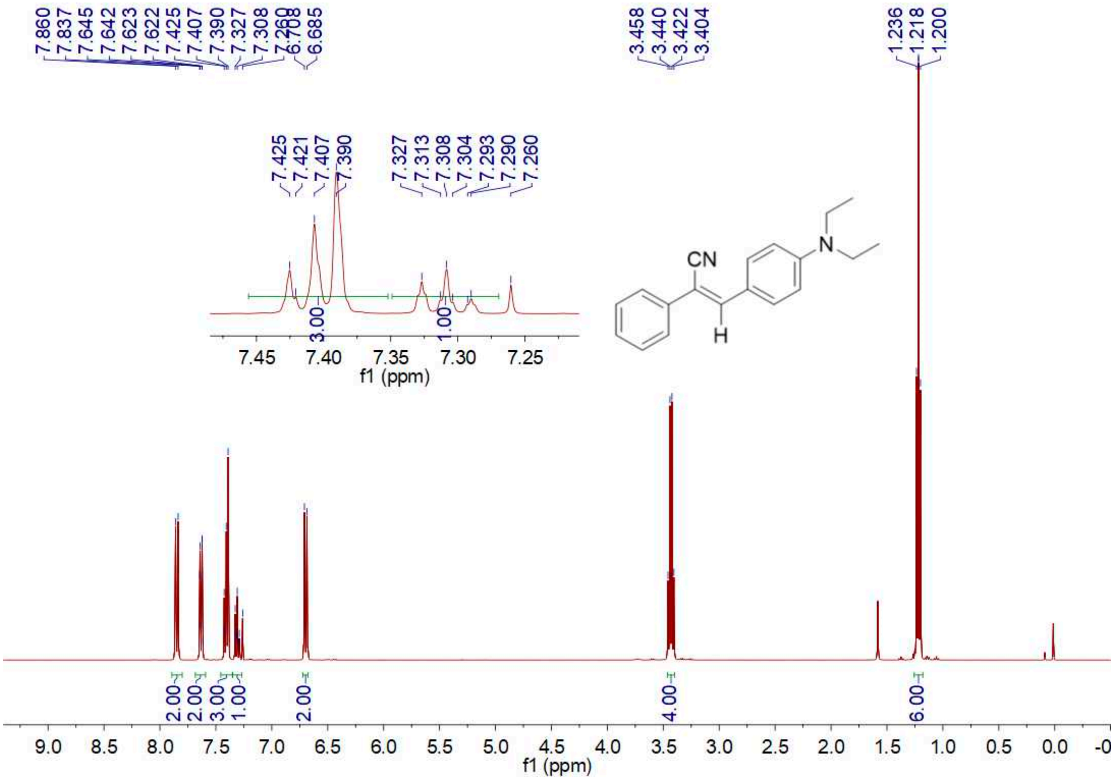

Figure S1. ^{1}H NMR spectra of **1** (in $CDCl_3$).

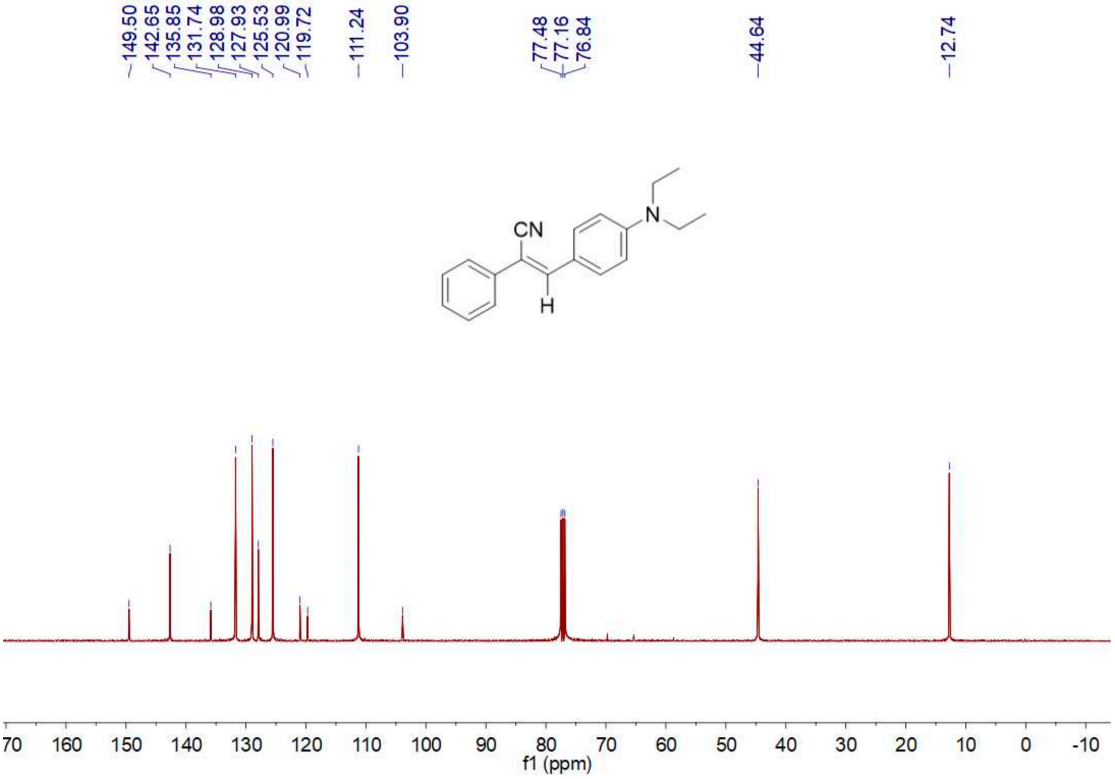

Figure S2. ^{13}C NMR spectra of **1** (in $CDCl_3$).

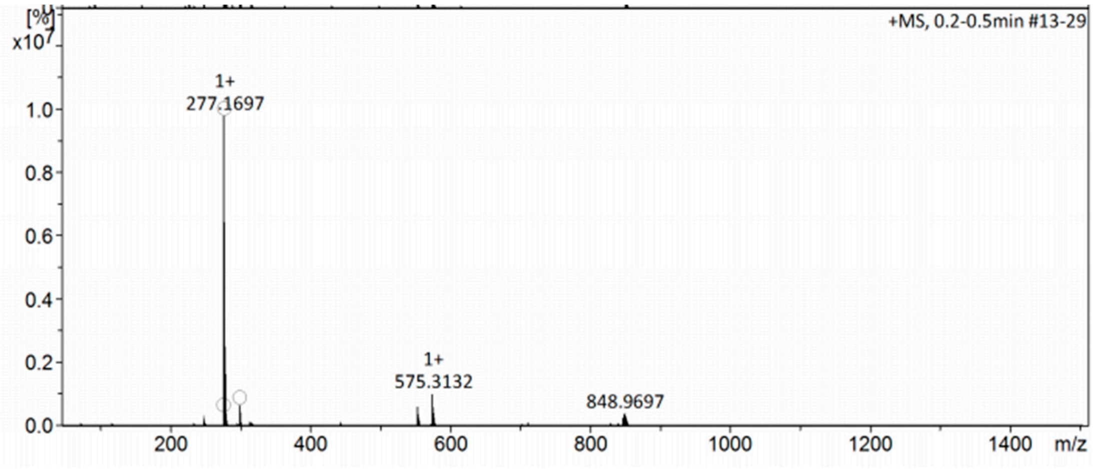

Figure S3. HRMS spectrum of **1**.

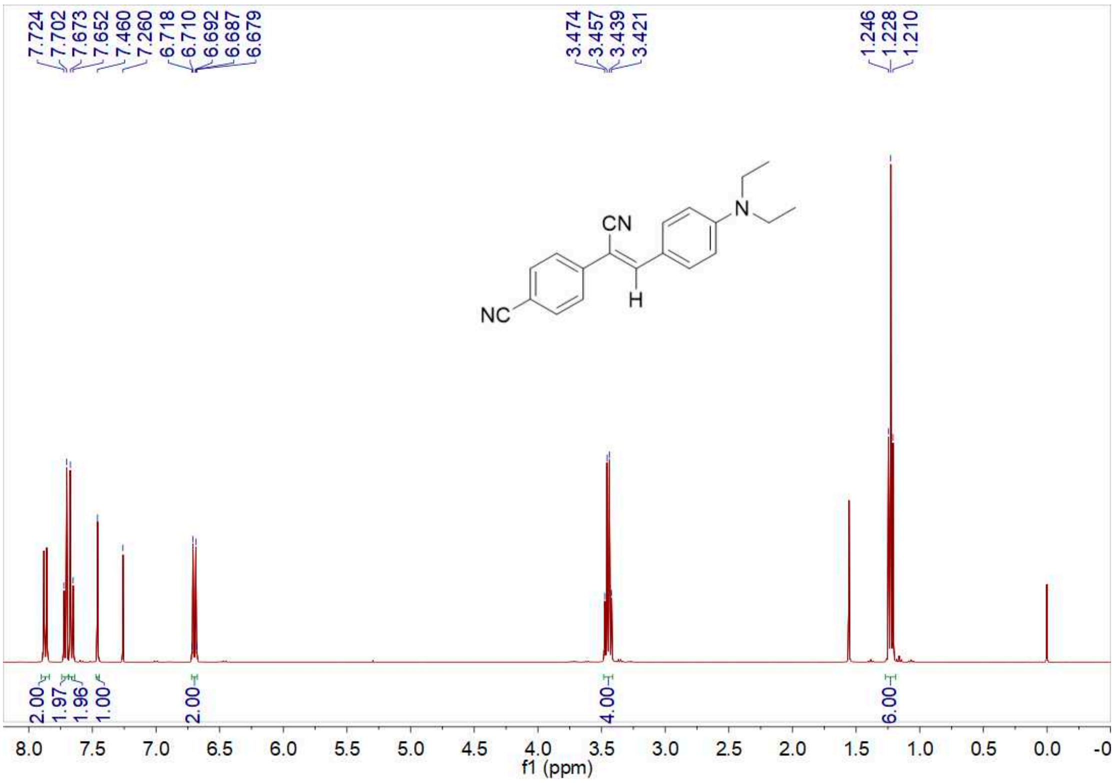

Figure S4 ^{1}H NMR spectra of **2** (in $CDCl_3$).

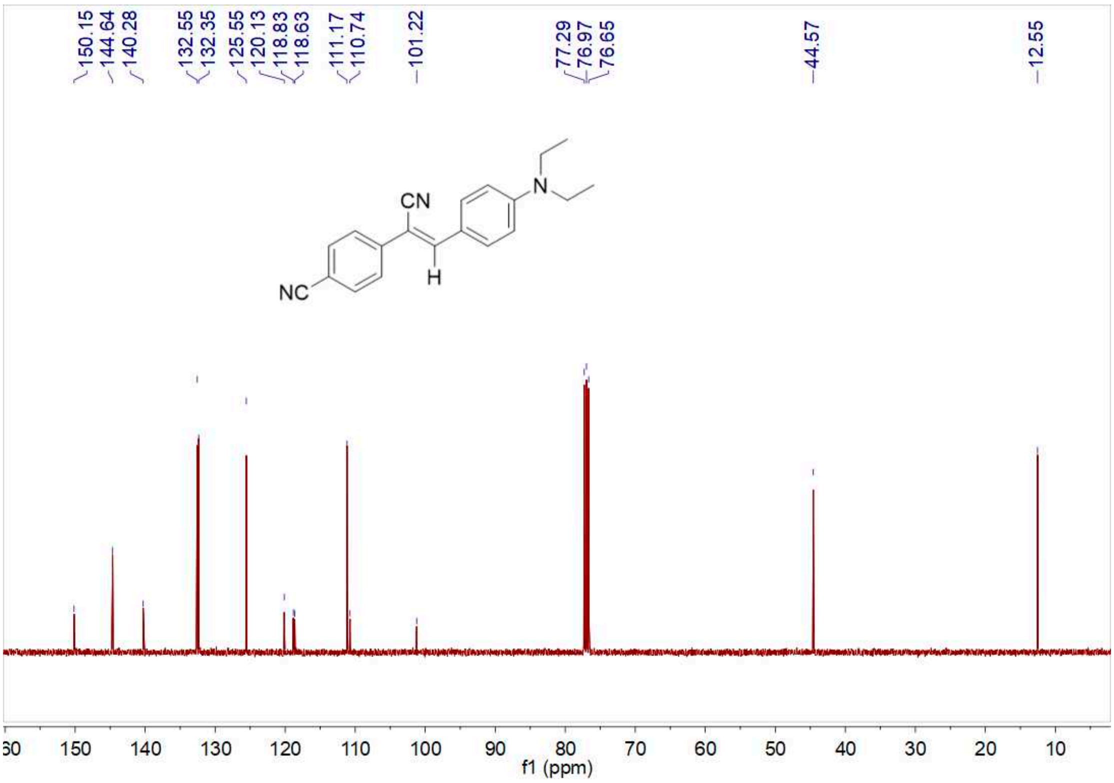

Figure S5. ^{13}C NMR spectra of **2** (in $CDCl_3$).

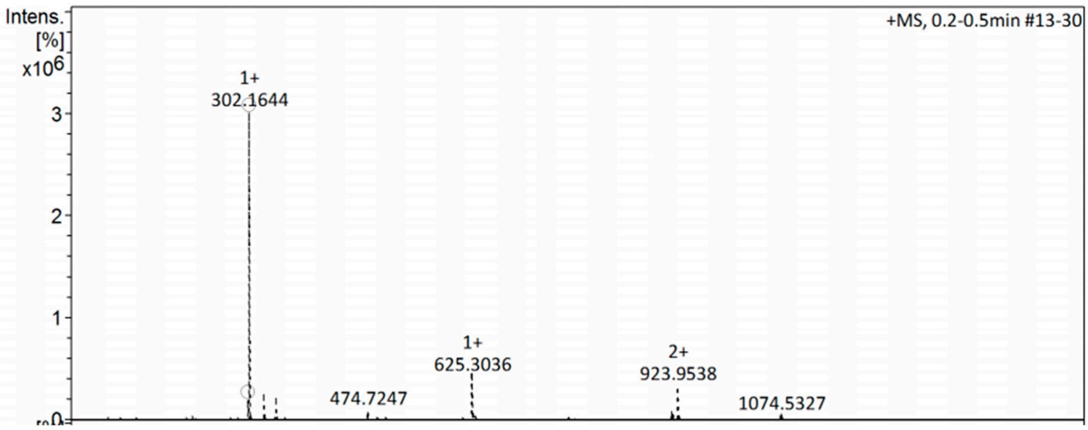

Figure S6. HRMS spectrum of **2**.

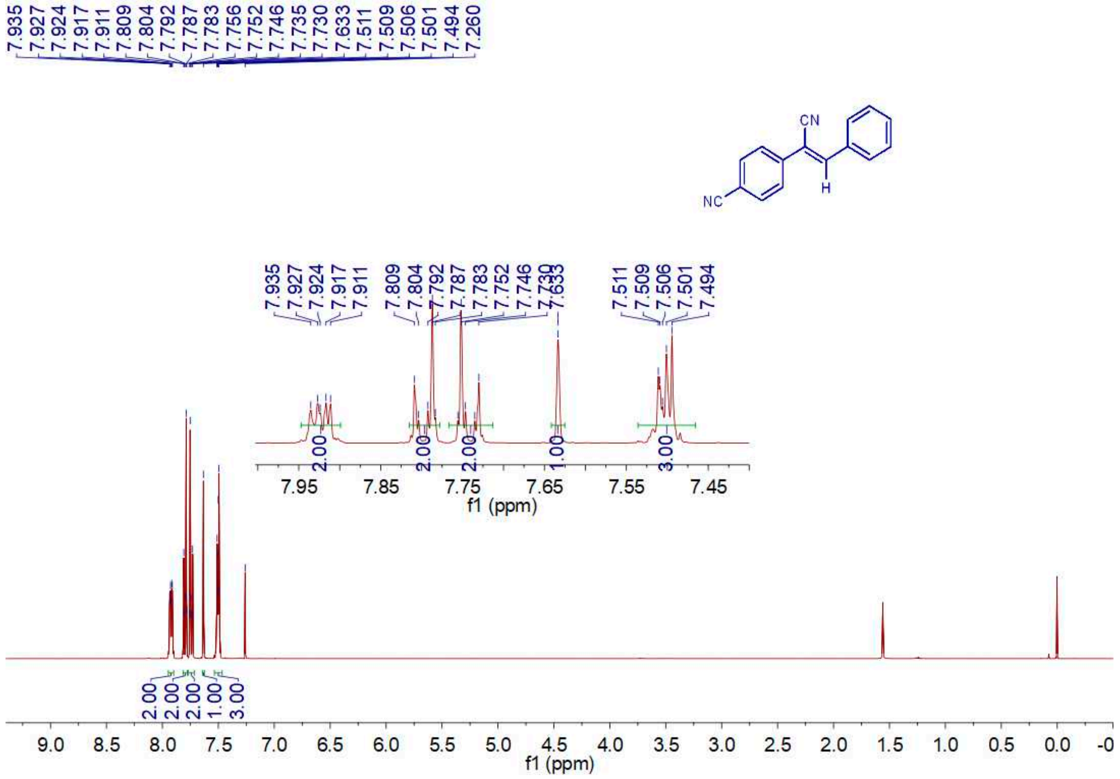

Figure S7. ^{1}H NMR spectra of **3** (in $CDCl_3$).

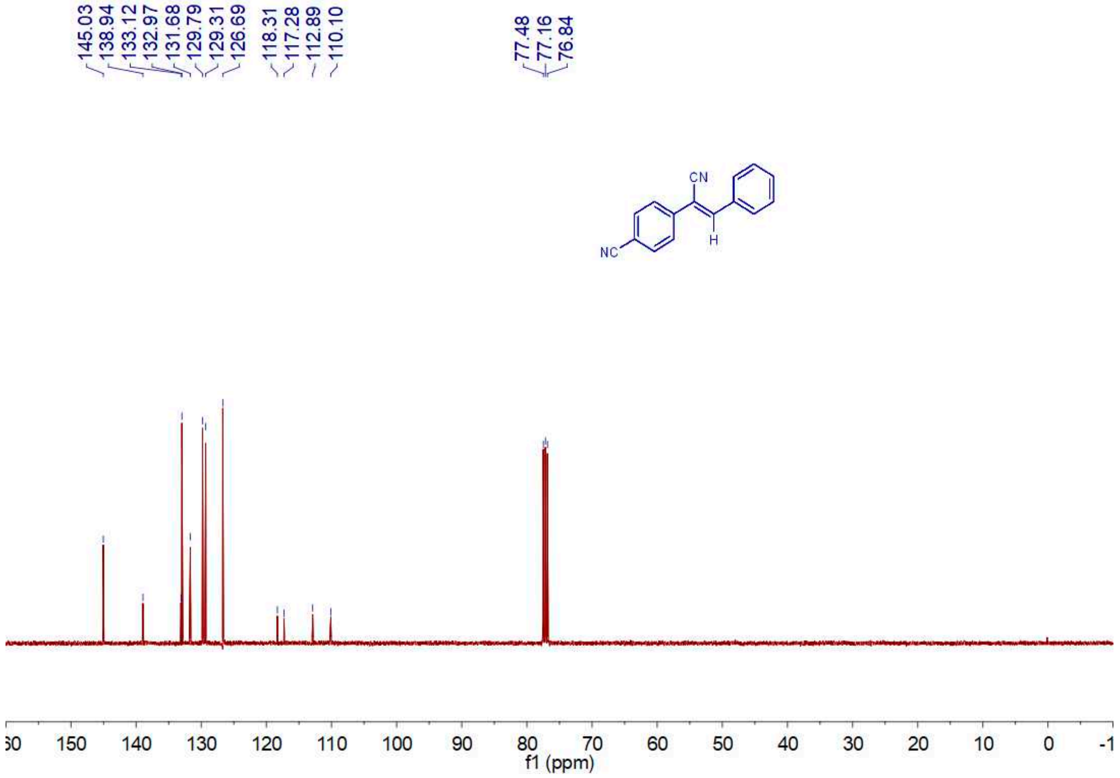

Figure S8. ^{13}C NMR spectra of **3** (in $CDCl_3$).

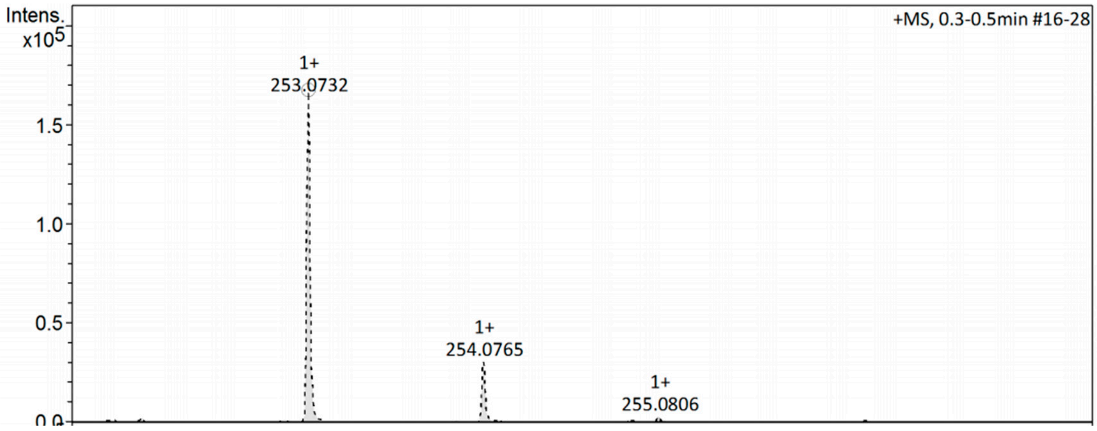

Figure S9. HRMS spectrum of **3**.

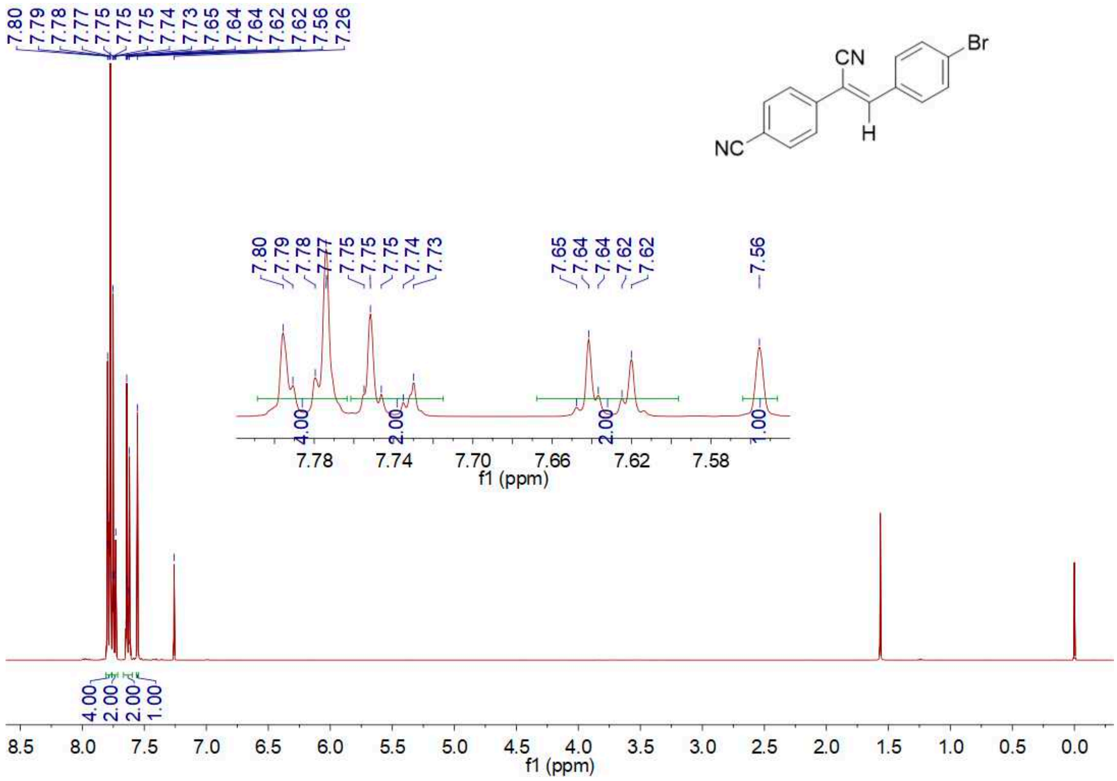

Figure S10. ^{1}H NMR spectra of **4** (in $CDCl_3$).

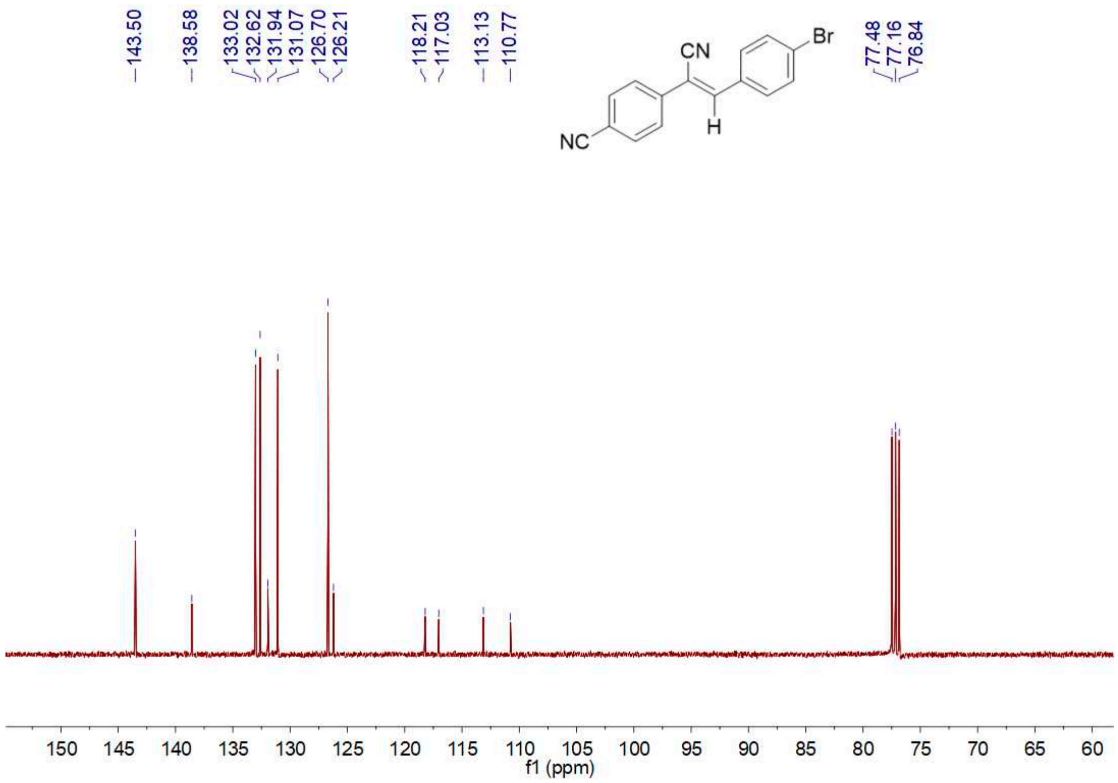

Figure S11. ^{13}C NMR spectra of **4** in $CDCl_3$.

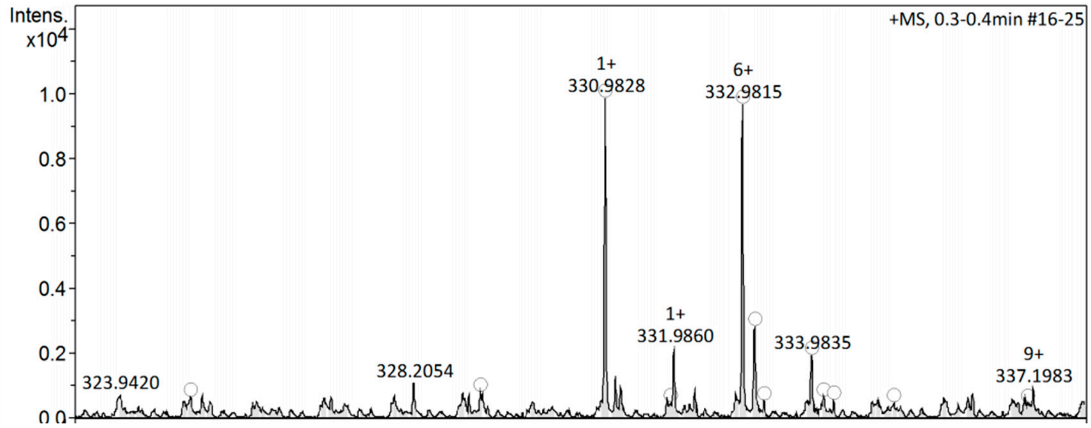

Figure S12. HRMS spectrum of **4**.

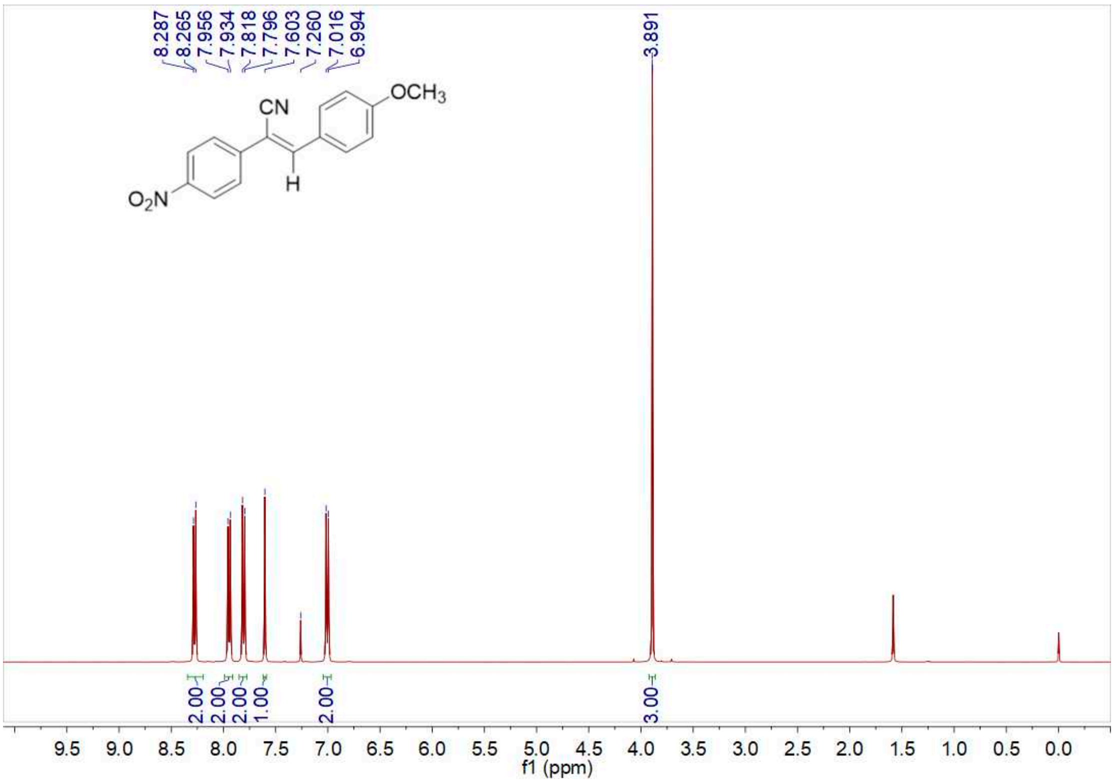

Figure S13. ^{1}H NMR spectra of **5** in $CDCl_3$.

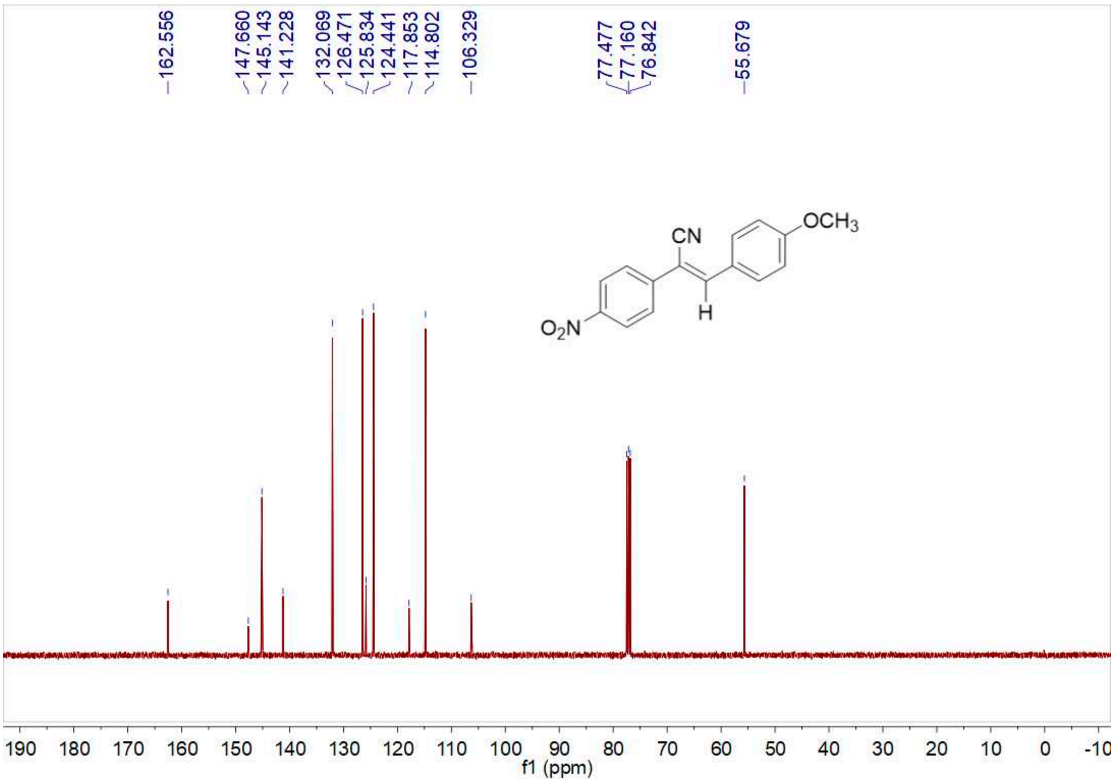

Figure S14. ^{13}C NMR spectra of **5** in $CDCl_3$.

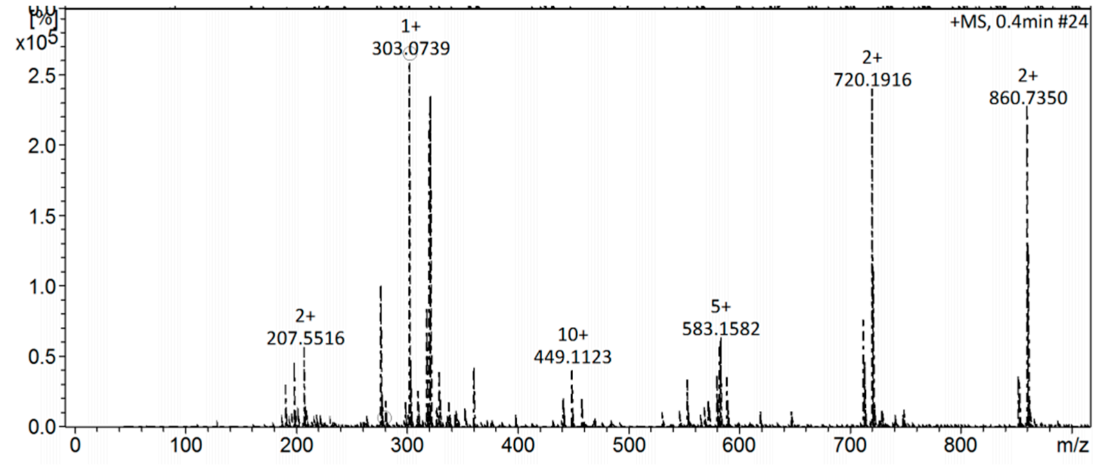

Figure S15. HRMS spectrum of **5**.

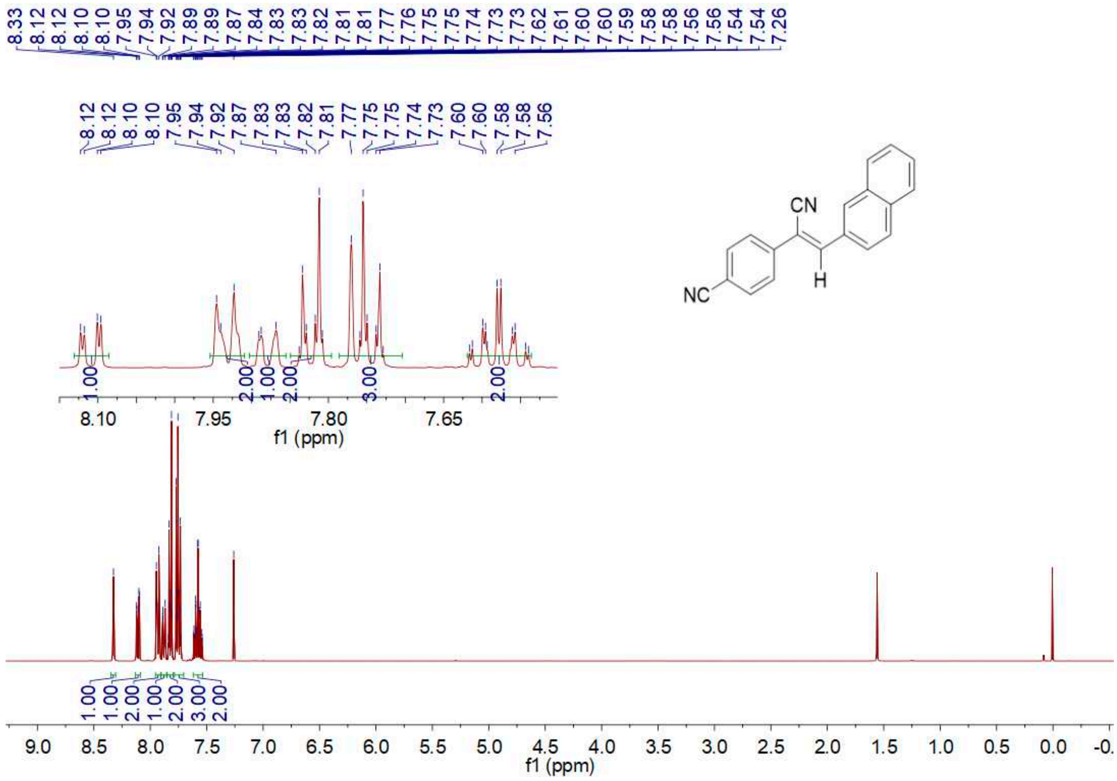

Figure S16. ^{1}H NMR spectra of **6** in $CDCl_3$.

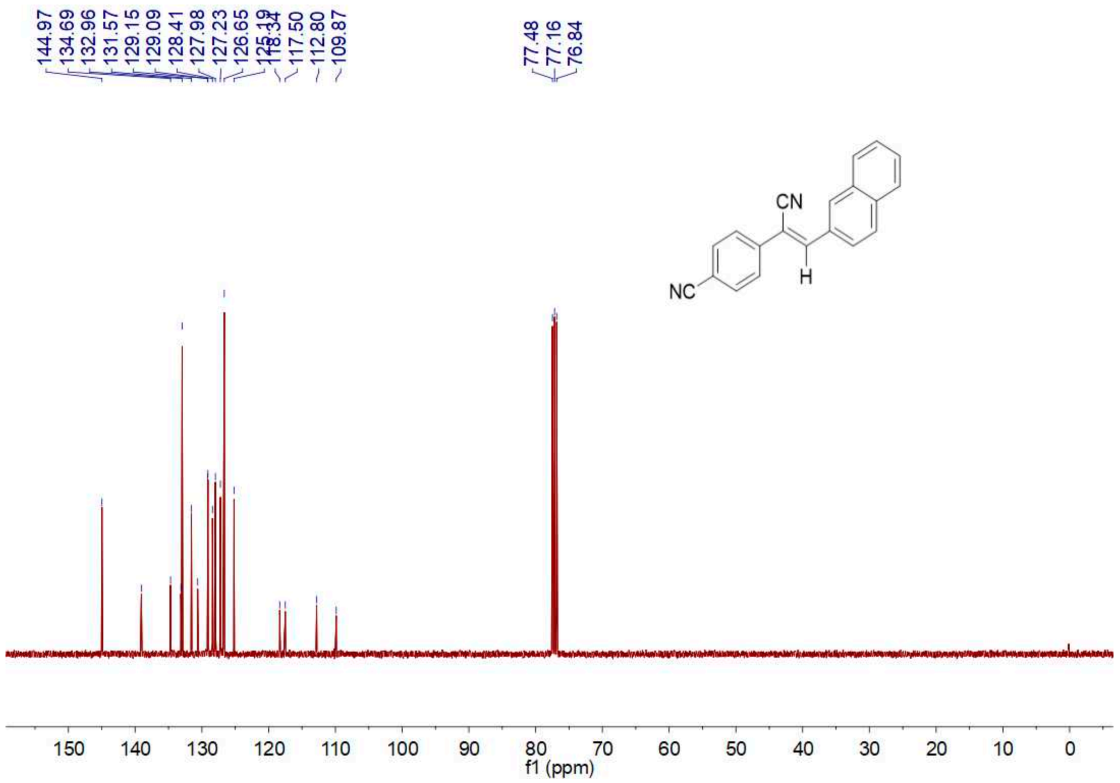

Figure S17. ^{13}C NMR spectra of **6** in $CDCl_3$.

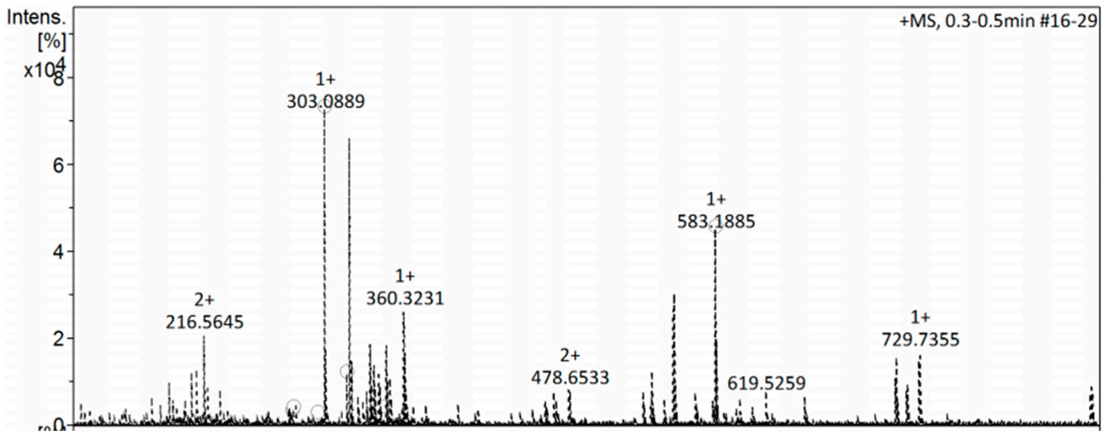

Figure S18. HRMS spectrum of **6**.

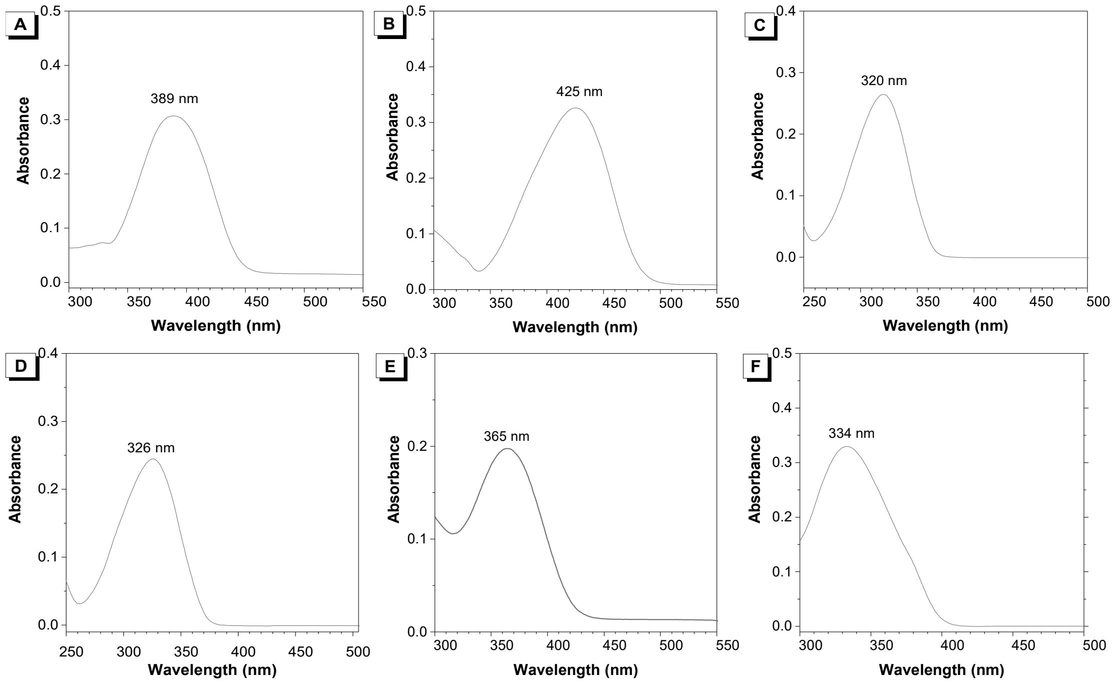

Figure S19. (A-F) Absorption spectra of molecules **1-6** in THF solution, c = 1 × 10^{-5} M.

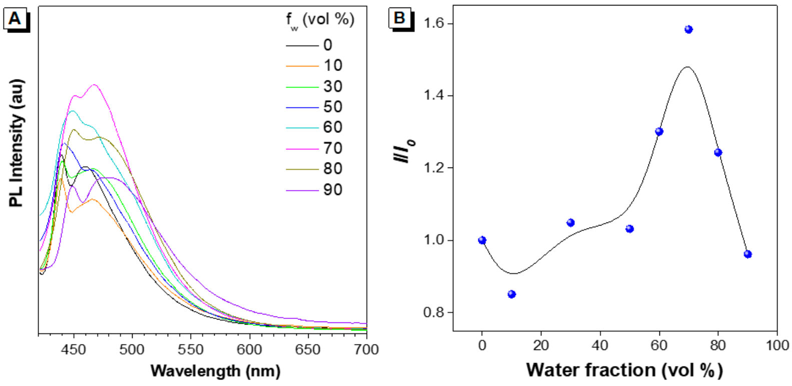

Figure S20. (A) PL spectra of **1** in THF and THF/water mixture with different water fractions. (B) PL intensity ratio of I/I_0 in different THF/water mixtures, c = 1 × 10^{-5} M.

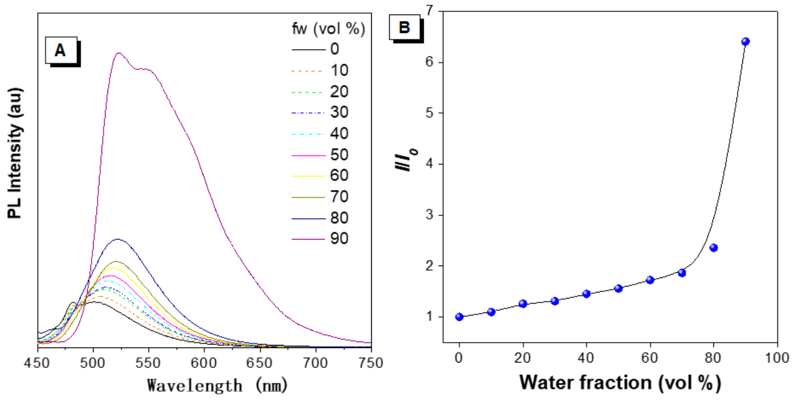

Figure S21. (A) PL spectra of **2** in THF and THF/water mixture with different water fractions. (B) PL intensity ratio of I/I_0 in different THF/water mixtures, c = 1 × 10^{-5} M.

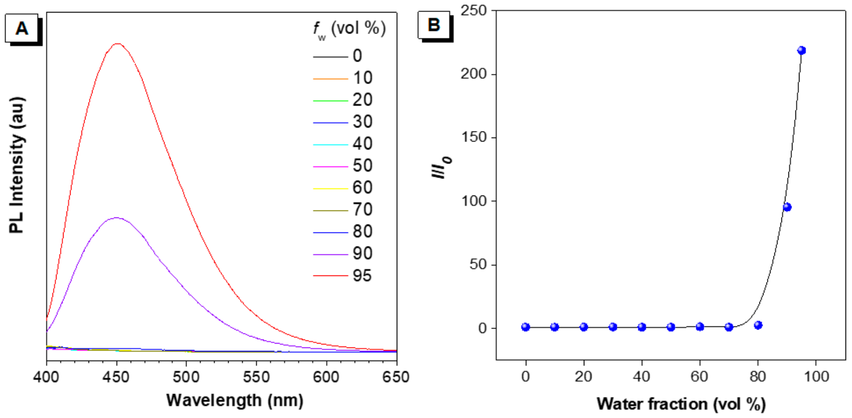

Figure S22. (A) PL spectra of **3** in THF and THF/water mixture with different water fractions. (B) PL intensity ratio of I/I_0 in different THF/water mixtures, c = 1×10^{-5} M.

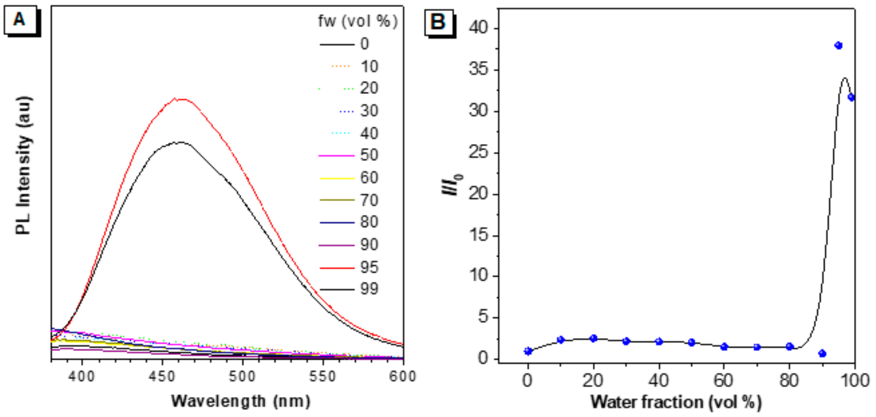

Figure S23. (A) PL spectra of **4** in THF and THF/water mixture with different water fractions. (B) PL intensity ratio of I/I_0 in different THF/water mixtures, c = 1×10^{-5} M.

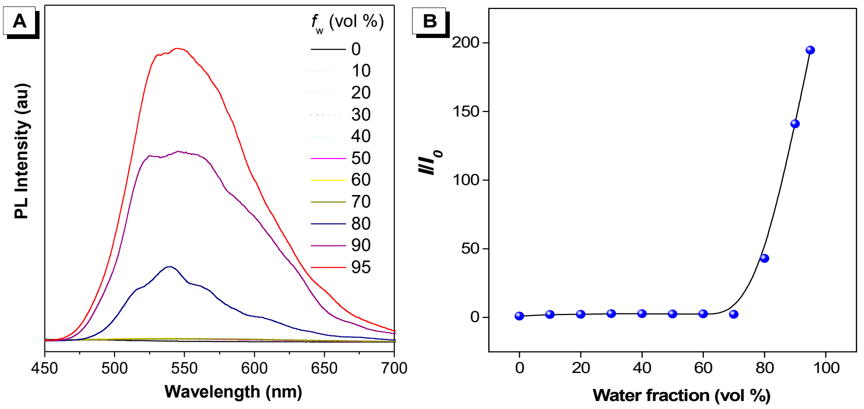

Figure S24. (A) PL spectra of **5** in THF and THF/water mixture with different water fractions. (B) PL intensity ratio of I/I_0 in different THF/water mixtures, c = 1×10^{-5} M.

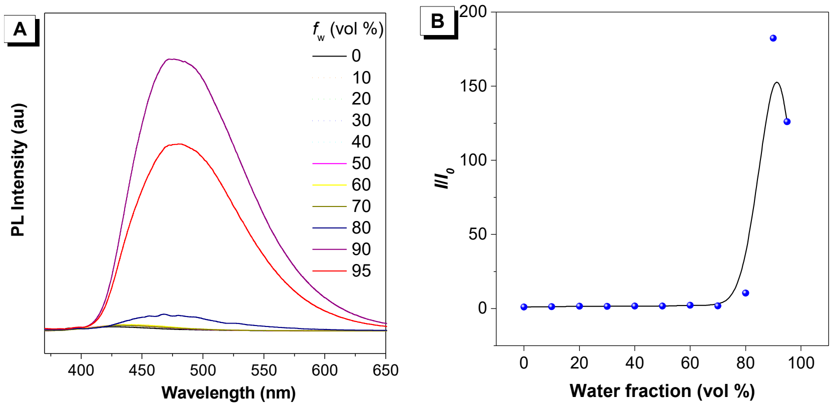

Figure S25. (A) PL spectra of **6** in THF and THF/water mixture with different water fractions. (B) PL intensity ratio of I/I_0 in different THF/water mixtures, c = 1×10^{-5} M.

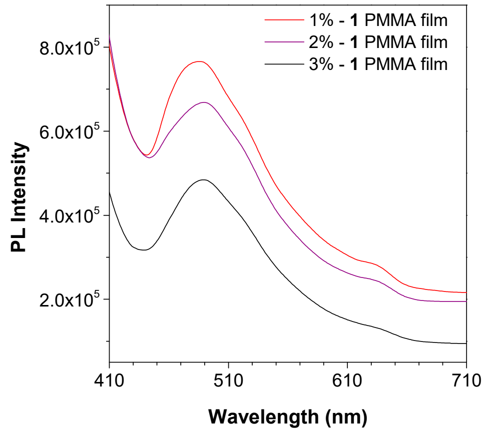

Figure S26. PL spectra of 1, 2, 3 wt% **1**-loaded PMMA film.

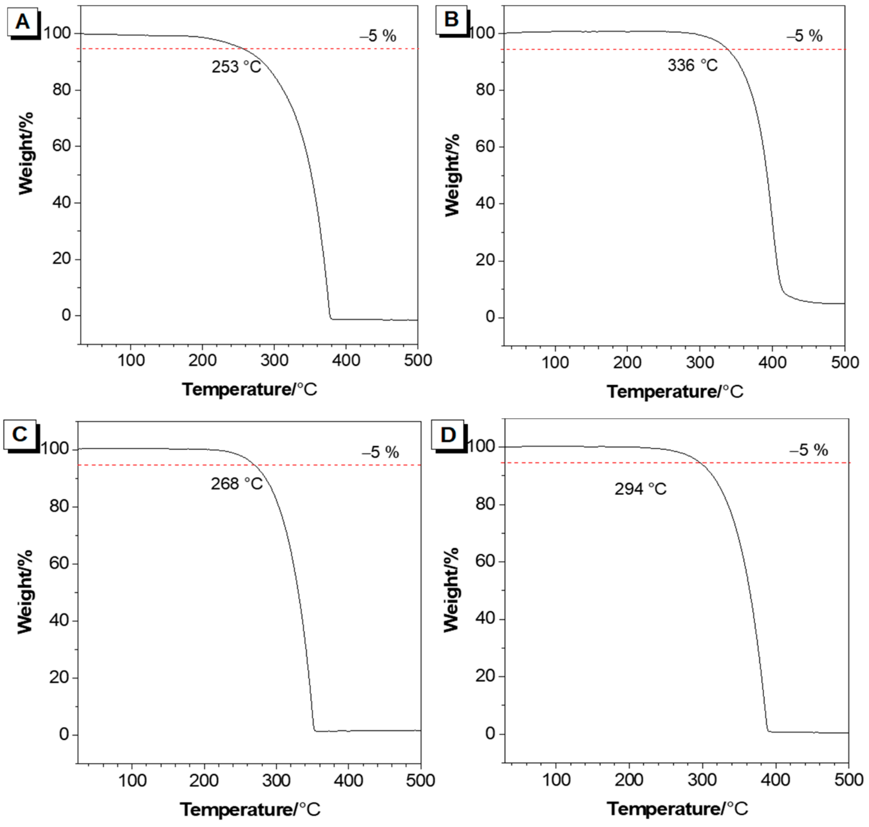

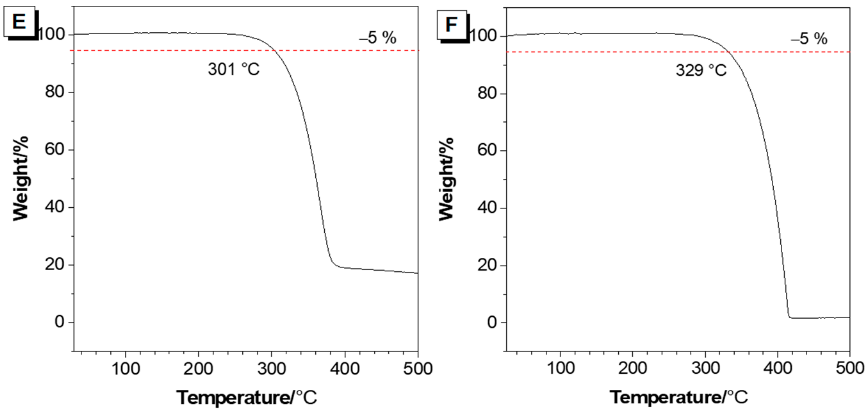

Figure S27. (A-F) Thermogravimetric analysis (TGA) of solids **1-6**.

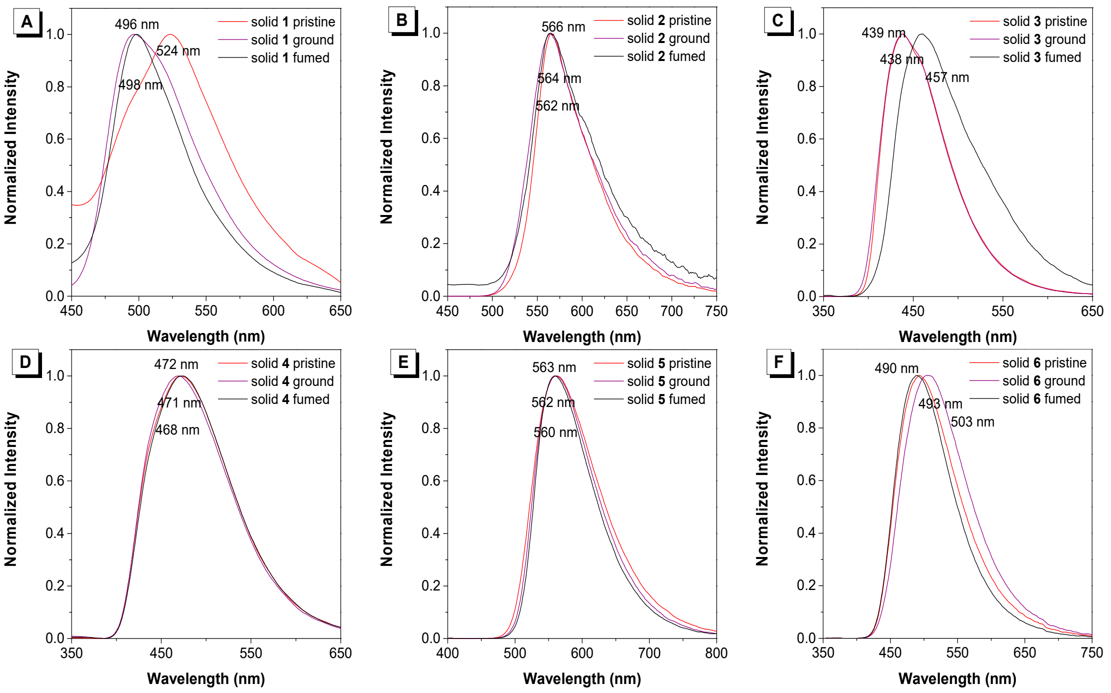

Figure S28. PL spectra of molecules **1-6** as pristine and ground solids. Molecule **1** (Excitation wavelength = 389 nm), molecule **2** (Excitation wavelength = 425 nm), molecule **3** (Excitation wavelength = 320 nm), molecule **4** (Excitation wavelength = 326 nm), molecule **5** (Excitation wavelength = 365 nm), molecule **6** (Excitation wavelength = 334 nm).

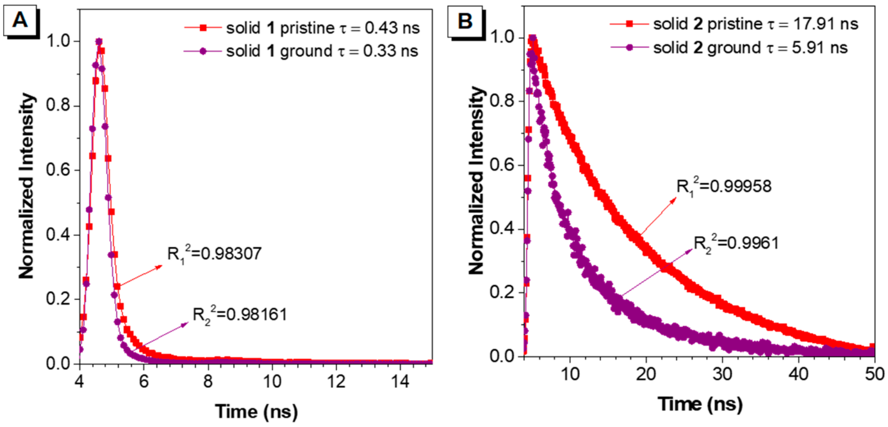

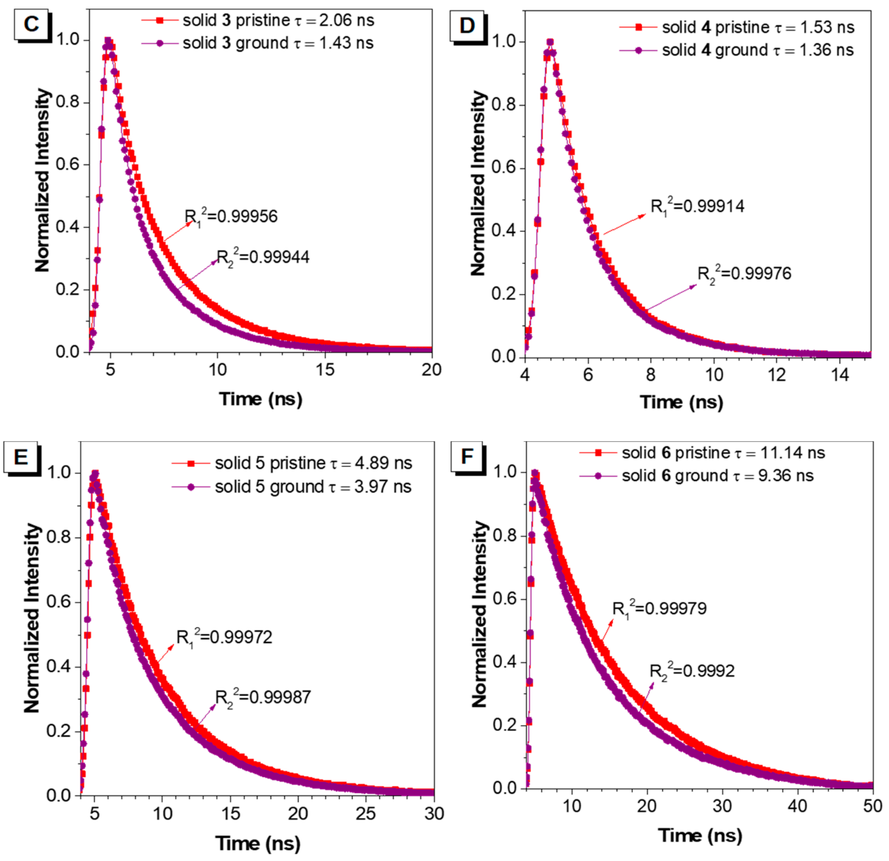

Figure S29. (A-F) Transient PL decay curves of solids **1-6** before and after anisotropic grinding.

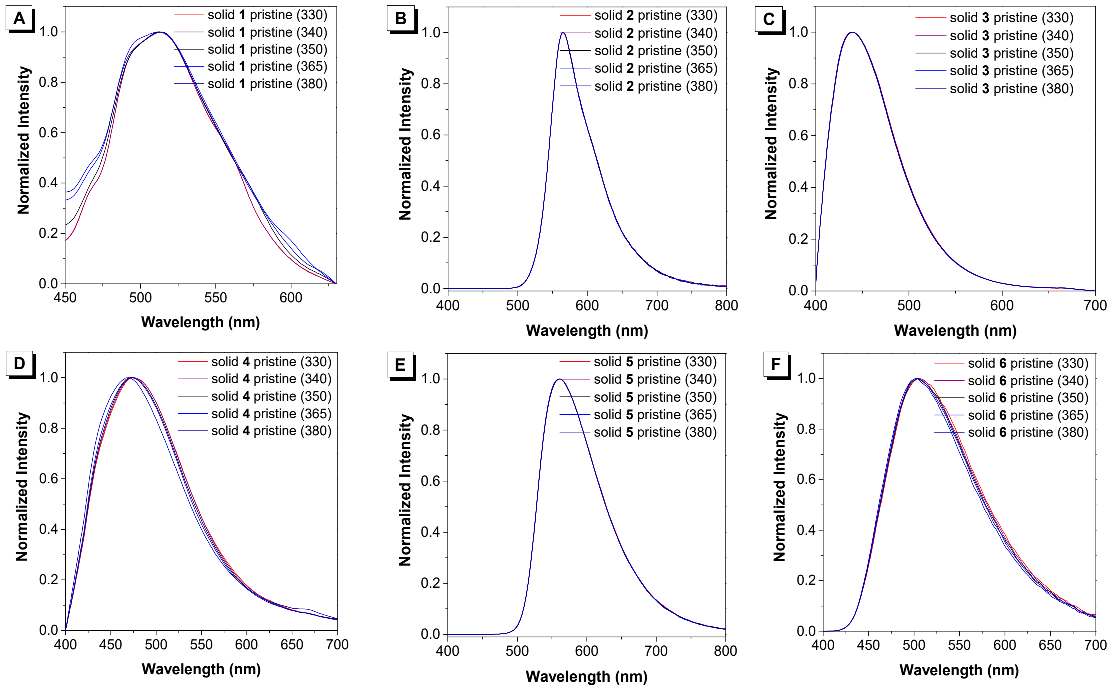

Figure S30. Normalized Intensity PL spectra of molecule **1-6** under the different excitation wavelength.

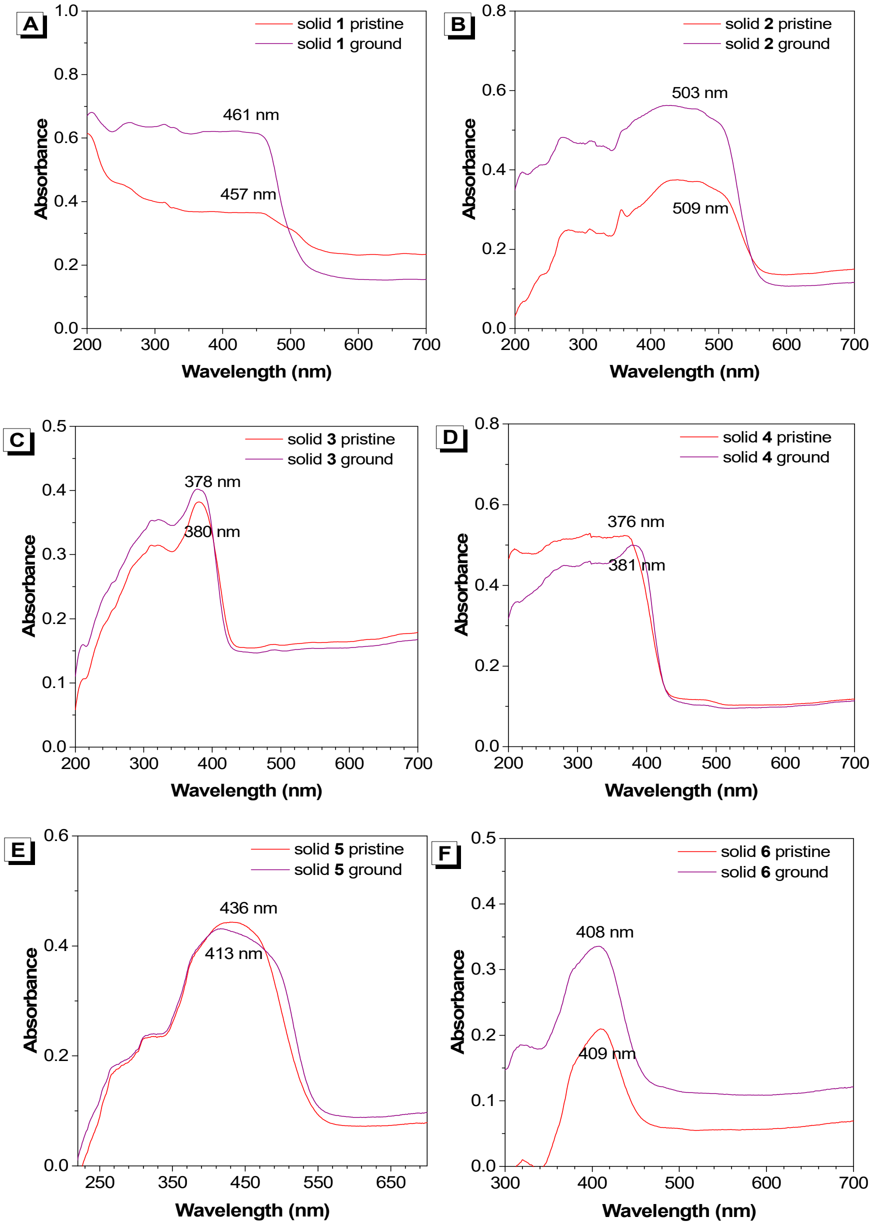

Figure S31. (A-F) UV-vis spectra of solids **1-6** before and after grinding.
